# Analyzing the Response Behavior of *Lumbriculus variegatus* (Oligochaeta: Lumbriculidae) to Different Concentrations of Copper Sulfate Based on Line Body Shape Detection and a Recurrent Self-Organizing Map

**DOI:** 10.3390/ijerph17082627

**Published:** 2020-04-11

**Authors:** Chang Woo Ji, Young-Seuk Park, Yongde Cui, Hongzhu Wang, Ihn-Sil Kwak, Tae-Soo Chon

**Affiliations:** 1 Fisheries Science Institute, Chonnam National University, Yeosu 59626, Korea; jichangwoo@gmail.com (C.W.J.); iskwak@chonnam.ac.kr (I.-S.K.); 2 Department of Biology and Department of Life and Nanopharmaceutical Sciences, Kyung Hee University, Seoul 02447, Korea; 3 Institute of Hydrobiology, Chinese Academy of Sciences, Wuhan 430072, China; ydcui@ihb.ac.cn (Y.C.); wanghz@ihb.ac.cn (H.W.); 4 Ecology and Future Research Association (EnFRA), Dusil-ro 45 beon-gil 21, Geumjeong-gu, Busan 46228, Korea

**Keywords:** *Lumbriculus variegatus*, line-tracking, response behavior, computational method, copper sulfate, risk assessment, Shannon entropy

## Abstract

Point detection (e.g., the centroid of the body) of species has been conducted in numerous studies. However, line detection (i.e., the line body shape) of elongated species has rarely been investigated under stressful conditions. We analyzed the line movements of an Oligochaeta *Lumbriculus variegatus* in response to treatments with a toxic chemical, copper sulfate, at low concentrations (0.01 mg/L and 0.1 mg/L). The automatic line-tracking system was devised to identify the movement of body segments (body length) and the movements of segments (i.e., the speed and angles between segments) were recorded before and after treatment. Total body length was shortened from 31.22 (±5.18) mm to 20.91 (±4.65) mm after the 0.1 mg/L treatment. The Shannon entropy index decreased from 0.44 (±0.1) to 0.28 (±0.08) after treatment. On the other hand, the body and movement segments did not significantly change after the 0.01 mg/L treatment. Sequential movements of test organisms were further analyzed with a recurrent self-organizing map (RSOM) to determine the pattern of time-series line movements. The RSOM made it feasible to classify sequential behaviors of indicator organisms and identify various continuous body movements under stressful conditions.

## 1. Introduction

Response behavior of indicator species is considered an efficient means of monitoring risk in aquatic ecosystems [1,2,3,4]. Real-time behavior measurements are able to fill the gap between macroscale (e.g., community surveys) and microscale (e.g., individual development) assessments [5,6]. Response behaviors of indicator species are sensitive to sublethal exposure of various toxic chemicals at low concentrations [1,7]. Response behaviors have been monitored in various taxa, including insects [8,9], crustaceans [2,10], snails [11], and fish [12,13,14].

Considering that indicator species are primarily either round (e.g., *Daphnia*), elongated, or fusiform (e.g., fish), point estimation methods (e.g., centroid of the body location) are conveniently used for behavioral monitoring [13,14,15]. However, it is also feasible to monitor species with long body shapes under stressful conditions, including oligochaetes [16] and nematodes (e.g., *Caenorhabditis elegans*) [17]. Compared with point movement, line movement provides an extra dimension to express the status of an indicator species. Line detection is primarily used with the nematode *Caenorhabditis elegans* [18,19,20]. *C. elegans* were also used to describe line movements of organisms by tracking the centroids of their *x* and *y* positions [21,22,23] and calculating the angle change rate of the body skeleton [19]. Line movements of *C. elegans* were used to differentiate between response behaviors of different mutants after exposure to benzene [24]. Computational methods, such as the Frenet–Serret formula, were also used to describe sequential line movements of *C. elegans* [20,25].

Recently, chemical engineering methods were developed to detect and remove copper ions from aqueous media [26,27,28]. These methods were relatively more convenient than behavioral monitoring methods. However, the potential environmental toxicity is not well characterized [29] and the risks they may pose to aquatic organisms and the environment are not certain. Oligochaeta, including *Lumbriculus variegatus*, are important fauna in the aquatic food chain or web and are widely used as bioindicators to pollution. In particular, *L. variegatus* was identified as an aquatic organism to assess test specimens of bioaccumulation by the US Environmental Protection Agency [30].

Although oligochaetes were used to indicate toxic effects [16,31,32], their line body shapes were not extensively studied or compared with those of *C. elegans*. Both nematodes and oligochaetes possess line body shapes, but their overall shapes are different. The body is more elongated in oligochaetes and the scope of movements is substantially more complex due to this body elongation. Although line movement shapes were not computationally analyzed for oligochaetes, various movement patterns were reported in response to stimuli. The body reverse and swimming movements of *L. variegatus*, for instance, were observed when the worms were touched by anterior segments [33]. The activities of Oligochaeta were recorded using a quadrupole impedance conversion technique under chemical stress [2,16,34], which showed an increase in locomotory activity of *L. variegatus* in an intermediate concentration of lead (1–5 mg/L) [16]. However, changes in body shapes of the oligochaetes per se were not extensively studied, especially in response to chemical stressors.

Regarding line body shape methodology, various computational approaches were introduced to analyze complex movement behaviors [13,15,35]. A multi-layer perceptron, which is a supervised artificial neural network, was used to detect changes in movement patterns of medaka fish (*Oryzias latipes*) before and after treatment with diazinon [15]. However, the multi-layer perceptron requires templates a priori to recognize the movement patterns in a supervised manner. Meanwhile, a self-organizing map (SOM), which is an unsupervised artificial neural network, was proposed to characterize movement patterns in an unsupervised learning manner (i.e., without a priori knowledge) and was implemented to classify response behaviors of various indicator species, including cockroaches [9] and medaka fish [13,14]. Son et al. [36] used a recurrent self-organizing map (RSOM), an extended version of a SOM, to pattern sequential data, in this case, time-series movements of *L. variegatus* to detect the effects of treatments with copper. Because of the complexity of behavioral data, one chemical concentration was primarily studied when analyzing movement patterns [13,15,37].

The temporal network models were more feasible in learning time-series data than conventional methods based on linear (e.g., Autoregressive Model and Autoregressive Moving Average-Neural Network) and non-linear (e.g., Nonlinear Autoregressive Moving Average with the Exogenous Input and Multivariate Adaptive Regression Splines) statistical analyses [38]. In ecology, time-series data for community dynamics were predicted using the Elman network [39] and a recurrent network [40]. These networks are necessary for training templates (i.e., supervised learning). However, obtaining all the pre-determined patterns for behavioral data is difficult due to the numerous complex patterns. Subsequently, a huge amount of behavioral data is required to be prepared through continuous recording. Data mining provides the overall scope of behavioral data; in this regard, we utilized the unsupervised network to accommodate the sequential line movement data by collecting data from continuous recording.

The Temporal Kohonen Map (TKM) [38], which was derived from the Kohonen Self-Organizing Map [41], is considered as an efficient learning tool for temporal sequence processing. The involvement of the earlier input vectors in each unit occurred following the recursive difference in TKM. RSOM and an unsupervised temporal model provided more flexibility when dealing with sequential data. In RSOM, direct learning of the temporal context is possible, while TKM is unable to directly use the temporal information of input sequences in weight updating [42]. TKM allows the use of many data with only a little a priori knowledge, while RSOM provides constant results to deal with the classification of temporal data with little effort [42,43,44].

In this study, we focused on evaluating the effects of exposure to chemicals on the body segments of *L*. *variegatus* at different concentrations using computational methods, including recurrent SOM. The body segments (i.e., body length), which were only obtained from line body shape detection, were characterized according to different concentrations of copper sulfate. We demonstrated that the unsupervised temporal model feasibly patterned the sequential line-movement of oligochaetes after toxic substance treatment and efficiently characterized the stressful behavior of the specimens.

## 2. Materials and Methods

### 2.1. Test Organisms and Observation System

*L. variegatus* were collected at the long-term field survey station, Beopgi (35°21′44″ N, 129°06′15″ E, Yangsan, Republic of Korea), in August 2006, and used as a stock population in the Laboratory of Ecology and Behavior System, Department of Biological Sciences, Pusan National University, Republic of Korea. The sample site was located upstream in the Suyong River and was free from anthropogenic effects due to the protection of the area by the local government for drinking water. The stock population was reared in a glass tank (5 L) with an artificial dry diet (Tetramin^®^) at 20 ± 1 °C. Dry leaves (oak, *Quercus* sp.) gathered at the sample site were used as the substrate in the tank. Only mature organisms that were 4 cm (±0.5 cm) in length were used for behavioral observations.

The movement behaviors of test specimens were individually observed and recorded using the observation system for 6 h without treatment. Subsequently, copper sulfate (CuSO_4_) was used as a treatment for the same organisms, which were recorded for an additional 6 h after treatment. After being dissolved in distilled water, the chemical was applied to the observation aquarium at two concentrations (0.01 and 0.1 mg/L). The LC_50_ of copper sulfate was reported as 0.23 mg/L [45]. The response behaviors of 20 individuals were observed before and after chemical treatments at each concentration using the observation system. After consideration of noise and observed data quality due to the elongated body shape in recorded images, 12 of 20 observed individuals at each concentration were selected for further analyses.

### 2.2. Detection of Line Movement

*L. variegatus* were individually placed in an observation aquarium (9 cm diameter) and their positions were scanned from the top view at 0.25 s intervals using a CCTV (Closed circuit television) camera (Kukjae Electronics Co. Ltd.; IVC-841). The time interval of 0.25 s was sufficiently short to observe the slow, overall responses of the line bodies of the oligochaetes. The backlight conditions (backlight; 0.2 Watt green diodes) were provided underneath the observation cage according to Son et al. [36] to enhance visual recognition of the organisms. Before placing the test organisms in the observation aquarium, a background image was acquired with an image size of 320 × 240 pixels. Then, a new image with the test organism in the observation aquarium was compared with the background image.

Considering the complexity in calculating line movements of animals with elongated body shapes [18,19], we adopted four processes to record the positions of test organisms before analyzing movements. First, we obtained line skeletons of the elongated bodies of the oligochaetes. Second, the heads and tails of the line skeletons were determined by experienced observers after watching continuous movement sequences. Due to the difficulty in automatic recognition of the head and tail in the elongated bodies of the oligochaetes, direct observations were performed to head and tail positions. For instance, the head initiated the movement and the rest of the body followed the head movement in a complex manner, with the last movement usually ending in a tail movement. Additionally, a wide range of turning was observed in the head area in a short period (broad angular speed). However, direct observation was time-consuming and some segments were difficult to identify as either the head or tail, particularly when test specimens performed reversal swimming, where the position of the head and the tail on the elongated body rapidly switched several times. When the body was released again, it was difficult to identify either the head or the tail. The segments with unclear identification were excluded from analysis, accounting for approximately 40% of observed data. Approximately 60% of the segments with activity were chosen for analysis. Third, the body skeleton was divided evenly into 12 segments. Based on the position of each segment, length (12 data points) and angles between segments (11 data points) were obtained at every time interval (0.25 s) for an average per 1 min (23 data points for one movement segment). Fourth, changes in body segments were investigated by calculating movement parameters under stressful conditions. The length of each segment and the curvatures between neighboring segments were recorded at each time interval by image framing according to Son et al. [36].

### 2.3. Analysis of Movement Data

Based on preliminary studies with test organisms [5,36], the following five parameters (average in 1 min) were selected to characterize the movement of the body segments of oligochaetes:

- Speed (mm/s): Average of movement distance of the test organisms in 1 min;
- Stop duration (s): Average time during which the test specimen did not move;
- Stop number: Stop frequency during 1 min;
- Turning rate (rad/s): The sum of angular changes in radians in absolute values divided by the cumulated time duration of movement;
- Meander (rad/s): The sum of angular changes in radians in absolute values divided by the cumulated movement distance.

In addition, the Shannon entropy index (*H*) [46] was calculated to represent diversity in movement activities in response to chemical stress, as follows:

$$H=-\sum_{i=1}^{n}\;p\left(x_{i}\right)\log_{2}p\left(x_{i}\right),$$

where *p(x_i_)* is the probability of observing the test organisms at each coordinate position (*i*). The entropy index represents the complexity of the movement activities of the test organisms. If the organisms were active with a higher degree of movement, the index was higher.

An analysis of variance (ANOVA) and Tukey’s honestly significant difference test were conducted to compare differences in variables among clusters. Statistical differences in the variables before and after chemical treatment were evaluated based on the paired sample *t*-tests. These analyses were conducted with the software Statistica [47].

Recurrence of time series data for movement was applied to the RSOM [42]. For time-series data, the sequential line data consisting of 12 segments with 23 data points (including 12 body segment lengths and 11 angles between segments) were used as input data for the RSOM. In total, data for 5 min (25 s × 12 = 300 s) were used to characterize the behavioral responses using the RSOM. Based on a previous study [36], we used 48 (=8 × 6) of the RSOM output units to show the most biologically relevant results.

The SOM is a vector quantization method to map patterns from an input space *V_i_* onto a lower dimensional space *V_M_* of the map to determine the best matching unit *b* in time step *t*, according to the following equation:

$$\left\|x(t)-w_{b}(t)\right\|=\min_{i}\{\left\|x(t)-w_{i}(t)\right\|\},$$

where *i* ∈ *V_M_*, *x*(*t*) is an input vector and *w_i_*(*t*) is a weight vector of the units *i* on the map. Then, the weight vector of the best matching unit b is updated toward the given input vector *x*(*t*) in the SOM, according to the equation:

(1) $$w_{b}(t+1)=w_{b}(t)+\gamma (t)h_{b}(t)(x(t)-w_{b}(t)),$$

where γ(t), 0<γ(t)≤ 1 is a learning rate and *h_b_*(*t*) is the neighborhood function.

RSOM is similar to SOM, except it searches for the best matching unit as following [36,42]:

(2) $$y_{i}(t)=(1-\alpha )y_{i}(t-1)+\alpha (x(t)-w_{i}(t)),$$

where 0 < α ≤ 1 is a leaking coefficient, *y_i_*(*t*) is a leaked difference vector, *w_i_*(*t*) is the reference or weight vector in unit *i,* and *x*(*t*) is the input pattern for time step *t*. The best matching unit *b* at time step *t* is searched for by the equation:

(3) $$y_{b}=\min_{i}\{\left\|y_{i}(t)\right\|\},$$

where *i* ∈ *V_M_*. The RSOM had the same weight updating process as the SOM, although the input sequence was noted before the learning process [36].

The learning process of the RSOM was conducted with a program developed by the authors in the Matlab environment [48]. After the learning process, a hierarchical cluster analysis was conducted based on the Ward linkage method with Euclidean distance measures to define the cluster boundaries in RSOM units. A multi-response permutation procedure [49], which is a nonparametric procedure to test the hypothesis of no difference between two or more groups, was conducted to evaluate the significance of the clusters defined in the RSOM using PC-ORD [50].

## 3. Results

### 3.1. Effects on Body Length and Parameters

**Figure 1:**
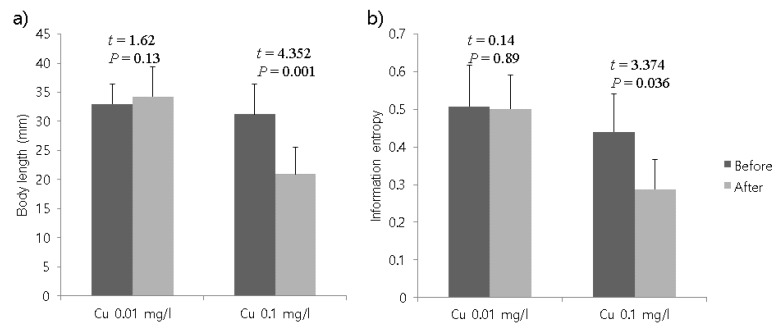
Comparison of total body lengths of *Lumbriculus variegatus* (**a**) and Shannon information entropy representing movement of test organisms (**b**) before and after treatments with copper sulfate at concentrations of 0.01 mg/L and 0.1 mg/L. Paired sample *t*-tests were conducted to compare the differences in body length and Shannon entropy index before and after treatments. Error bars indicate standard deviation.

The differences in the average body length of the organisms were evaluated before and after treatment with copper sulfate. Total length was not different before and after treatments at the 0.01 mg/L concentration (paired *t*-test, *t* = 1.62, *P* = 0.13), whereas it was significantly shortened from 31.22 ± 5.18 (S.D.) mm to 20.91 ± 4.65 (S.D.) mm after the 0.1 mg/L treatment (paired *t*-test, *t* = 4.352, *P* = 0.001) (Figure 1a).

The Shannon entropy index was not differentiated when the test organisms were treated with the 0.01 mg/L concentration; the values were 0.508 ± 0.11 (SD) before treatment and 0.501 ± 0.09 (SD) after treatment (paired *t*-test, *t* = 0.14, *P* = 0.89) (Figure 1b). However, the Shannon entropy index significantly decreased after the 0.1 mg/L treatment (0.44 ± 0.1 (SD) before treatment and 0.287 ± 0.08 (SD) after treatment), although the difference was not statistically significant (paired *t*-test, *t* = 3.674, *P* = 0.036) (Figure 1b). Considering the overall decrease in the entropy value, movement activities of test individuals became less diverse after treatment with 0.1 mg/L of copper.

**Figure 2:**
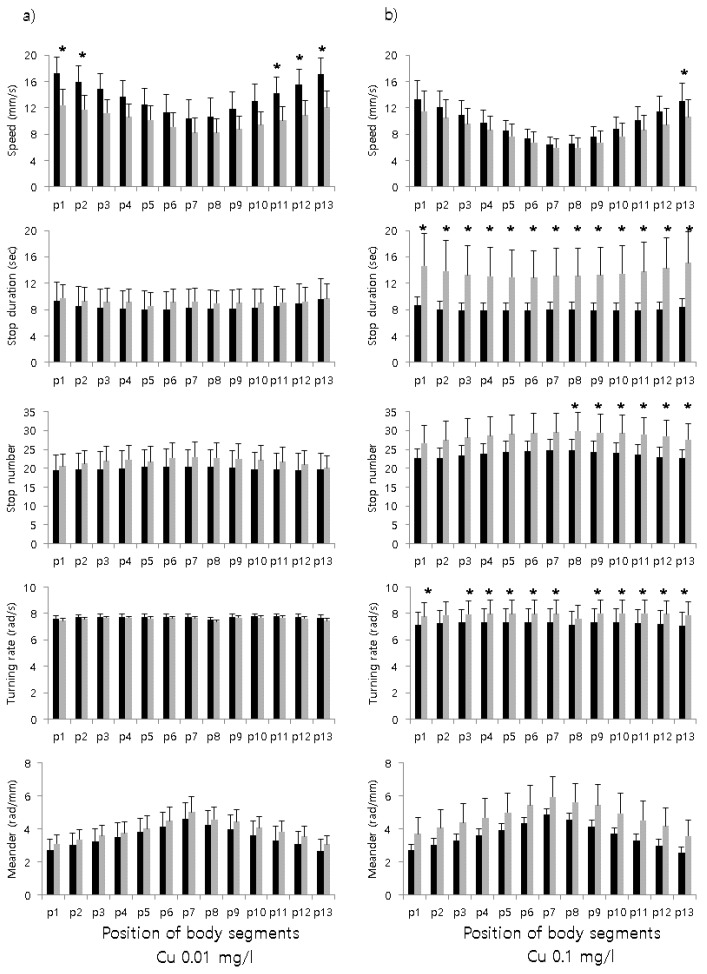
Differences in parameters characterizing the movement of body segments of *Lumbriculus variegatus* before and after copper sulfate treatments at two concentrations, 0.01 mg/L (**a**) and 0.1 mg/L (**b**). Statistical differences (paired sample *t*-test, *t*_0.05,(2),11_ = 2.20) in the selected variables are represented by an asterisk (*: 0.01 < *P* < 0.05). Error bars indicate standard deviation.

Subsequently, five parameters were evaluated according to the segments of line-shaped bodies before and after treatment at the two concentrations (Figure 2). Overall, speed was lower in the middle part of the body (top panel, Figure 2), whereas the meander was higher in the middle part of the body (bottom panel, Figure 2) before and after treatments, regardless of concentration. Meander tended to be lower at both ends of the body, although this difference was not statistically significant (Figure 2).

The chemical effects were observed more clearly with 0.1 mg/L compared with 0.01 mg/L. Stop duration and turning rate substantially increased in all or most segments at 0.1 mg/L, respectively (Figure 2). Additionally, a significant increase in stop number was observed in the posterior segments. It was noteworthy that only the last segment showed a significant decrease in speed after treatment at 0.1 mg/L. At the lower concentration of 0.01 mg/L, the difference was not as clearly observable as in the 0.1 mg/L concentration. However, a partial difference was observed in speed according to body segments, with a decrease in both the anterior (p1 and p2) and posterior segments (p11–p13) (paired *t*-test, *P* < 0.05; top panel, Figure 2a), indicating that a body response was observed at the lower concentration. According to ANOVA and Tukey’s honestly significant difference test (Table 1), speed and turning rate were divided into two groups, whereas stop duration and meander were not different between the experimental conditions.

The overall speed was lower in the middle parts of the body. Speed and meander showed high correlations at both concentrations (r = −0.815 and −0.915, respectively, for 0.01 mg/L and 0.1 mg/L concentrations; *P* < 0.0001 for both concentrations), indicating that the linear and circular movements were sensitive to the chemical but negatively associated with each other. Based on the results of body size (Figure 1) and parameters obtained from body segments (Figure 2, Table 1), the overall effects of treatments appeared to be distinctive at the 0.1 mg/L concentration and partially detectable in speed within the body shape at the lower concentration of 0.01 mg/L.

### 3.2. Effects on Angles of Body Segments

**Figure 3:**
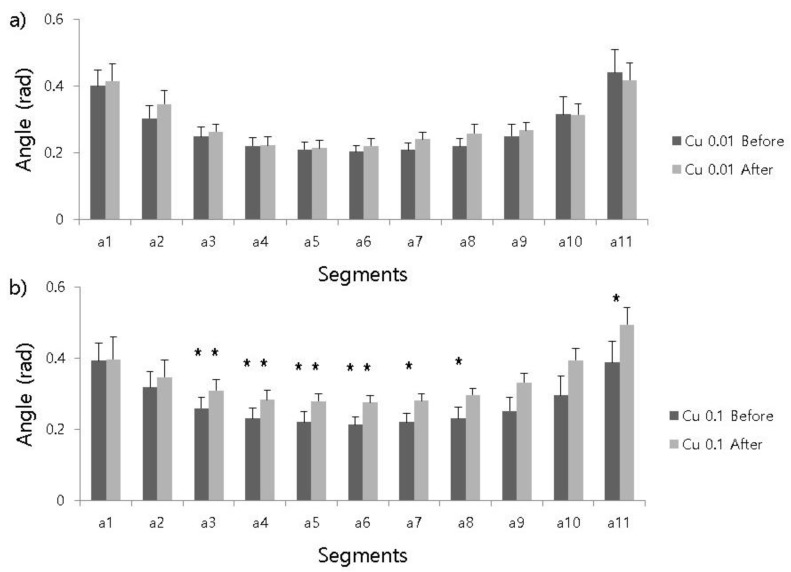
Differences in curvatures of the head (a1) and tail (a11) at two different concentrations of copper sulfate: (**a**) 0.01 mg/L and (**b**) 0.1 mg/L. Head and tail regions were identified by direct observation. Statistical differences (paired sampled *t*-test, *t*_0.05,(2),11_ = 2.20) in the curvatures are represented by an asterisk (*: *P* < 0.05; **: *P* < 0.01). Error bars indicate standard deviation.

The curvature of the body was represented by a series of angles observed between every consecutive pair of body segments, creating an extra dimension of movement patterns. The patterns of angle changes were different in the different concentrations (Figure 3). The curvature of the head (a1) and tail (a11) varied according to treatment strength of copper, as shown for both the 0.01 mg/L (Figure 3a) and 0.1 mg/L (Figure 3b) concentrations. Curvature strongly differed in the middle of the body at 0.1 mg/L. The curvature of the tail (a11) was sensitive to increases after the 0.1 mg/L treatment (0.389 ± 0.033 and 0.494 ± 0.088 for before and after treatments, respectively) (paired *t*-test, *t* = 2.247, *P* = 0.046). Curvatures were not different before or after the 0.01 mg/L treatment in any segment, including the head and tail segments (paired *t*-test, *t* = 0.573 and 0.503, *P* = 0.577 and 0.624 for head and tail, respectively). Curvature specifically at the head segment (a1) was not significantly different between the before (0.393 ± 0.046) or after 0.1 mg/L treatment groups (0.397 ± 0.055) (paired *t*-test, *t* = 0.091, *P* = 0.929).

### 3.3. Patterning Sequential Line Movement

**Figure 4:**
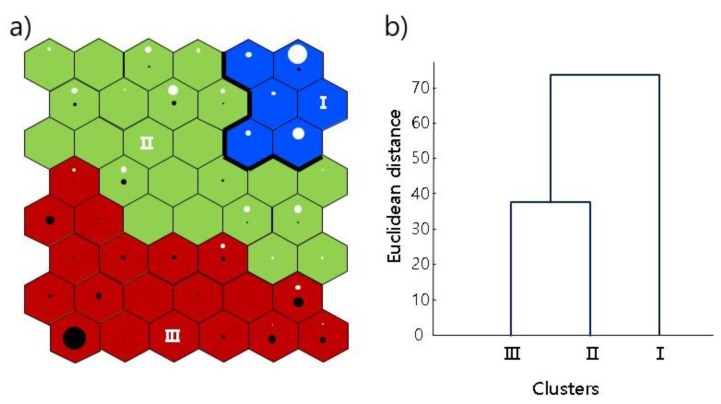
Recurrent self-organizing map (RSOM) applied to the line movements of *Lumbriculus variegatus* before (◯) and after (∙) copper sulfate treatments. (**a**) Classification of line movement tracks in the RSOM and (**b**) dendrogram for the classification of RSOM units based on the Ward linkage method with Euclidean distance measures. Different sizes of circles indicate the number of patterns assigned to each SOM unit.

Because the line movement was recorded in time series, continuous movements were patterned before and after chemical treatment via RSOM. Considering the effects of the chemical were conspicuous at high concentrations (Figure 1 and Figure 2), we used data for the 0.1 mg/L copper sulfate treatment to pattern the sequential line movement. The movement patterns were accordingly grouped using RSOM (Figure 4a). Different sizes of circles indicated the number of patterns assigned in each SOM unit. A gradient was observed in the vertical position with a slight inclination to the diagonal. The movement patterns were grouped vertically into three clusters (I, II, and III) (Figure 4b). The before-treatment groups (white circles) were mainly placed in the upper right part of the map, whereas the after-treatment groups (black circles) occupied a broader area in the bottom left part of the map. The movement patterns before treatment were mainly grouped in cluster I, whereas the movement patterns after treatment were grouped in cluster III.

**Figure 5:**
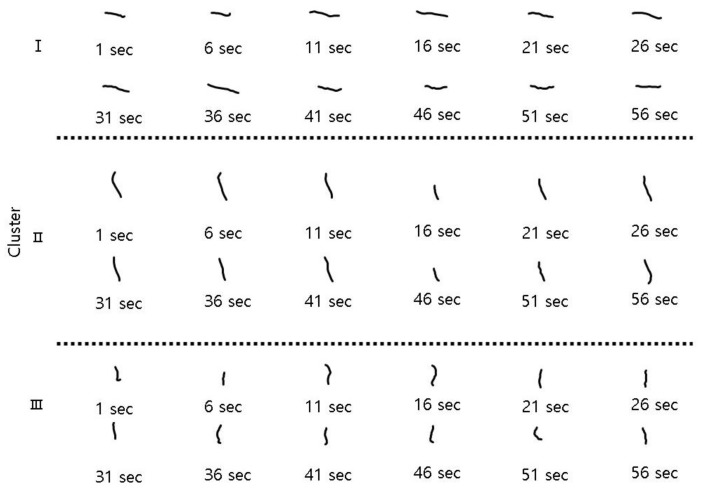
The sequential tracks of line movements of test organisms in different clusters defined in the RSOM. The number underneath each figure indicates the legend of a snapshot at 5 s intervals.

The body movements were correspondingly different in different clusters (Figure 5). Cluster I showed a sequence of large and straightened shapes, cluster II displayed folding shapes, and cluster III represented contracted and folding shapes. As stated above, body shapes in cluster II were mostly movements before the chemical treatment, whereas those in cluster III were mostly movements observed after the treatment. Therefore, body shapes after the chemical treatments were represented by short body lengths with a high degree of folding.

**Figure 6:**
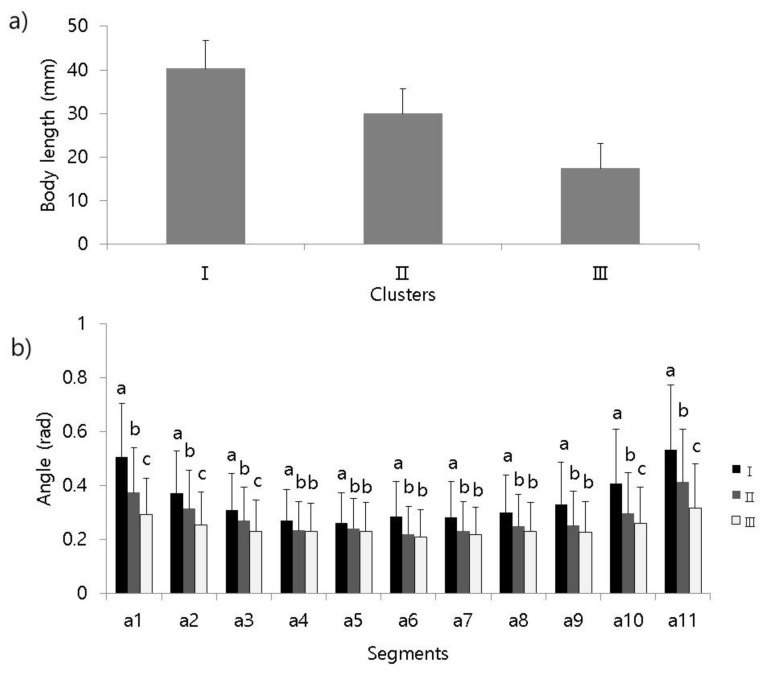
Variation in body segments of *Lumbriculus variegatus* according to subclusters matching the RSOM. (**a**) Total length and (**b**) angle between body segments. Error bars indicate standard deviation. Different letters on error bars indicate significant differences between clusters based on Tukey’s honestly significant difference test (*P* < 0.05).

Each movement parameter differed according to different clusters in the RSOM (Figure 6). To compare parameters, 1000 samples were randomly selected from each cluster. The total length in cluster I showed the greatest range among the clusters (Figure 6a), with length also being relatively greater in cluster II, another cluster belonging to the before-treatment group, compared with cluster III, which represented after treatment. The total lengths of each cluster significantly differed from each other (Tukey’s test, q_0.05,3000,4_ = 3.314; I ≠ II ≠ III) (Table 2). The angles between body segments were also significantly different among clusters. In cluster I, the angles were relatively greater at the end of the body, however, curvatures were greater in the middle part of the body. Patterns in the RSOM were capable of characterizing the sequential line shapes of test organisms before and after treatment.

## 4. Discussion

We demonstrated that the movement of segments of line-shaped animals (e.g., oligochaetes) could be analyzed to detect chemical stress at low concentrations. Detailed differences could be seen in the body segments (Figure 1 and Figure 3). Differences in response behaviors were also observed according to different toxin concentrations (Figure 2), Shannon entropy indices (Figure 1b), and angles (Figure 3), demonstrating that response behaviors were associated with the strength of the toxic impact. It was also noteworthy that differences in responses were represented by differences in angles according to different toxin concentrations. Although angle changes were higher at the center positions at higher concentrations with regard to the total body, the angle changes were differentiated at the end positions at low concentrations. Greater curvature was observed at the center position, indicating sinuousness of the body (Figure 3). However, parameters representing speed in body segments were not sufficiently differentiated to detect stress behaviors of test organisms.

Although detection of the response behaviors based on body segments may require extra computational resources (e.g., calculating body length and curvature) than movement parameters, body segments appeared to be efficient in determining detailed response behaviors, including partial movements of line-shaped bodies. The angle of the tail significantly changed after treatment with the toxic substance, indicating that the tail behavior of test organisms was more affected than the head region (Figure 3b). Line-shaped body visualization would be useful to characterize line-movement patterns of test organisms due to the extra dimension included to express the complexity of response behaviors compared with point data.

We used *L. variegatus* as the test organism in this study. Aquatic oligochaetes, including *Lumbriculus*, are important taxa in freshwater communities. Oligochaetes play an important role in aquatic communities, aiding in the decomposition of organic materials in sediment. In particular, *L. variegatus* is sensitive to exterior stimuli and has a diverse scope of behaviors [33,51], and was proposed as a standard organism for sediment bioaccumulation tests [52]. This organism has stereotypical behaviors that can be used for sublethal toxicology [16,53,54], and could be a biological indicator. The species was reported to be more sensitive to toxic chemicals, including copper, compared with other indicator species in Tubificidae, such as *Tubifex tubifex* [55]. However, line movements were not reported after exposure to stressors prior to this work. To the best of our knowledge, the pattern analysis of line data for oligochaetes, as shown in this study, was not previously conducted in response to toxic chemicals. Time-series analysis by RSOM could be a stepping-stone for the quantitative patterning of continuous movements of elongated animals, such as oligochaetes.

We used copper sulfate as a toxicant in this study. Copper is commonly used for fertilizers and pesticides due to its antifungal properties; however, excessive levels can contaminate aquatic ecosystems, even though it is an essential trace element [56]. Monitoring and removal methods were developed due to its toxicity to organisms [57,58]. Regarding efficiency in chemical engineering tools, biomonitoring with observations of response behaviors were not able to measure the precise concentration of toxic materials. However, the risk of environmental toxicity of copper is varied in regard to freshwater organisms. We demonstrated that copper sulfate caused typical symptomatic behaviors in the temporal sequence of line movement of blackworms according to different concentrations. The toxic effects were mostly reported at one concentration in continuous behavioral monitoring [5,13,15]. However, physiological–behavioral studies are needed in the future to reveal the causality of relationships in the production of symptoms.

Line tracking of test specimens is typically performed with *C*. *elegans* to characterize movement patterns according to different phenotypes [18,20,22]. Although *C. elegans* is a good indicator for the analysis of behavior according to phenotype, some drawbacks exist regarding monitoring in practical situations. This species is somewhat small and a microscope is usually required for monitoring. Consequently, it would be difficult for monitoring under field conditions, requiring more in situ facilities. In addition, the nematode’s life-span is too short for continuous monitoring under real conditions. However, aquatic Oligochaeta, including *Lumbriculus,* are reasonable species for monitoring under field conditions because of their large size and long life-span.

In this study, the head and tail regions were not specifically differentiated through digital image processing. We manually identified the head and tail regions of oligochaetes in movement segments, and modified methods were used to confirm body parts. Feng et al. [22] distinguished the heads and tails of *C. elegans* manually on a monitor. In the preliminary study, we attempted to automatically identify the head and tail regions of *C. elegans*, but it was difficult to automatically distinguish between the head and tail regions of oligochaetes. Because the body was much longer and movement behaviors of oligochaetes (e.g., body reverse swimming) were highly complex compared with those of *C. elegans*, further development of methods to recognize body parts in elongated animals, such as oligochaetes, is required in the future.

## 5. Conclusions

Analysis of the body segment data of the line-shaped animals was useful for detecting responses. Line-shaped body movements with parameters expressing body segments broadened the scope of behavioral characterization of elongated animals, adding an extra dimension to reveal the complexity of line-shaped bodies compared with point detection in round-shaped species. RSOM is therefore helpful in addressing the sequential line movements of test organisms, especially line-shaped animals.

## Figures and Tables

**Table 1:** Tukey’s multiple comparison test for parameters between two concentrations (a: before 0.01 mg/L; b: after 0.01 mg/L; c: before 0.1 mg/L; d: after 0.1 mg/L). Groups are divided by upper lines and under lines based on Tukey’s multiple comparison test (*P* < 0.05).

Position	Speed	Stop Duration	Stop Number	Turning Rate	Meander
p1	n.s *.	n.s.	n.s.	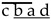	n.s.
p2	a $\bar{bcd}$	n.s.	n.s.	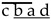	n.s.
p3	a $\bar{bcd}$	n.s.	n.s.	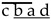	n.s.
p4	a $\bar{bcd}$	n.s.	n.s.	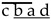	n.s.
p5	a $\bar{bcd}$	n.s.	n.s.	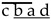	n.s.
p6	a $\bar{bcd}$	n.s.	n.s.	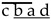	n.s.
p7	a $\bar{bcd}$	n.s.	n.s.	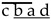	n.s.
p8	a $\bar{bcd}$	n.s.	n.s.	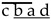	n.s.
p9	a $\bar{bcd}$	n.s.	n.s.	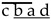	n.s.
p10	a $\bar{bcd}$	n.s.	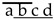	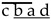	n.s.
p11	a $\bar{bcd}$	n.s.	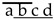	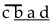	n.s.
p12	a $\bar{cbd}$	n.s.	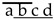	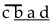	n.s.
p13	a $\bar{cbd}$	n.s.	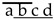	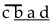	n.s.

* n.s.: statistically not significant

**Table 2:** Tukey’s multiple comparison test for homogeneous groups defined in the self-organizing map (SOM) for angles characterizing body segments of *Lumbriculus variegatus* before and after 0.1 mg/L and 0.01 mg/L copper treatments.

	**a1**	**a2**	**a3**	**a4**	**a5**	
F	100.89	35.16	25.16	10.60	5.09	
*P*	<0.0001	<0.0001	<0.0001	<0.0001	0.0056	
Group	I ≠ II ≠ III	I ≠ II ≠ III	I ≠ II ≠ III	I ≠ II = III	I ≠ II = III	
	**a6**	**a7**	**a8**	**a9**	**a10**	**a11**
F	33.82	22.26	21.03	40.61	55.64	70.96
*P*	<0.0001	<0.0001	<0.0001	<0.0001	<0.0001	<0.0001
Group	I ≠ II = III	I ≠ II = III	I ≠ II = III	I ≠ II = III	I ≠ II ≠ III	I ≠ II ≠ III
